# Interaction between sex and left ventricular reverse remodeling and its association with outcomes after transcatheter aortic valve implantation

**DOI:** 10.1007/s10554-022-02596-x

**Published:** 2022-04-21

**Authors:** Jurrien H. Kuneman, Steele C. Butcher, Jan Stassen, Gurpreet K. Singh, Stephan M. Pio, Frank van der Kley, Nina Ajmone Marsan, Juhani Knuuti, Jeroen J. Bax, Victoria Delgado

**Affiliations:** 1 Department of Cardiology, Leiden University Medical Center, Albinusdreef 2, 2300 RC Leiden, The Netherlands; 2 Turku PET Centre, University of Turku and Turku University Hospital, Turku, Finland; 3 Turku Heart Centre, University of Turku and Turku University Hospital, Turku, Finland

**Keywords:** Aortic stenosis, Left ventricular reverse remodeling, Sex differences, Transcatheter aortic valve implantation, Echocardiography

## Abstract

Women with severe aortic stenosis (AS) have better long-term prognosis after transcatheter aortic valve implantation (TAVI) compared to men. Whether this is caused by sex-related differences in left ventricular (LV) reverse remodeling after TAVI is unknown. Patients with severe AS who underwent transfemoral TAVI between 2007 and 2018 were selected. LV dimensions, volumes, and ejection fraction (LVEF) were assessed by transthoracic echocardiography before TAVI and at 6 and 12 months follow-up after TAVI. LV reverse remodeling was defined as the percentual LV mass index (LVMi) reduction compared to baseline. The primary outcome was all-cause mortality. A total of 459 patients (80 ± 8 years; 52% male) were included. At 6 and 12 months follow-up, both sexes showed significant reductions in LV volumes and LVMi accompanied by improvement in LVEF, without significant differences between the sexes over time. During a median follow-up of 2.8 [IQR 1.9–4.3] years, 181 (39%) patients died. Women showed better outcomes compared to men (log-rank p = 0.024). In addition, male sex was independently associated with all-cause mortality in multivariable Cox regression (HR 1.423, 95% CI 1.039–1.951, p = 0.028). No association was observed between the interaction of percentual LVMi reduction and sex with outcomes (p = 0.64). Men and women with severe AS had similar improvement in LVEF, and similar reductions in LV volumes and LVMi at 6 and 12 months after TAVI. Women showed better survival after TAVI as compared to men. The superior outcomes noted in women after TAVI are not associated with sex differences in LV reverse remodeling.

**Graphical abstract:**

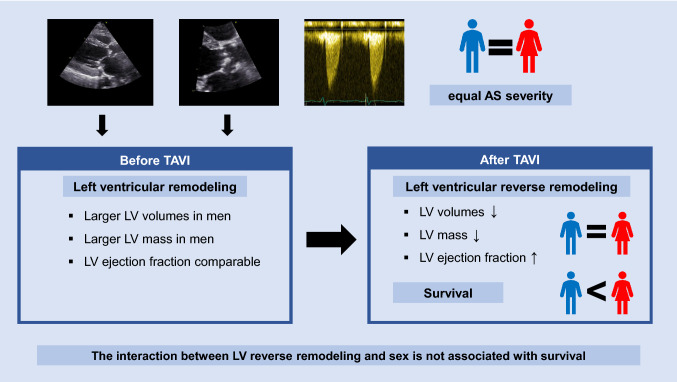

**Supplementary Information:**

The online version contains supplementary material available at 10.1007/s10554-022-02596-x.

## Introduction

Untreated severe aortic stenosis (AS) has a poor prognosis [1, 2]. Aortic valve replacement is an effective therapy and significantly reduces morbidity and mortality in patients with severe AS [3, 4]. Transcatheter aortic valve implantation (TAVI) has emerged as a valid alternative to surgical aortic valve replacement for patients with AS who are inoperable or have high operative risk [5]. Moreover, recent studies showed promising results of TAVI in patients at intermediate and low surgical risk [6–8]. Interestingly, the survival benefit of TAVI differs between men and women [9–11]. Women with severe AS have shown an increased risk of complications during TAVI, but have better survival after TAVI compared to men [12, 13].

Differences in left ventricular (LV) reverse remodeling between men and women may be a potential explanation for the sex-related outcome differences after TAVI. The LV remodeling pattern in response to the pressure overload caused by severe AS may differ between men and women [14, 15]. However, little is known on how the regression of LV mass and changes in LV geometry occur after TAVI.

Some studies suggested the presence of sex-related differences in LV reverse remodeling after TAVI, including more LV mass regression and greater improvement in LV ejection fraction (LVEF) in women versus men [16–19]. However, the association between the different LV reverse remodeling patterns after TAVI between men and women as well as the differences in outcomes have not been fully elucidated. Accordingly, the aims of this study were to examine (1) sex differences in LV reverse remodeling after TAVI and (2) to evaluate whether these differences are associated with the different outcomes after TAVI.

## Methods

### Study population

Patients with severe AS treated with TAVI at the Leiden University Medical Center (Leiden, The Netherlands) between 2007 and 2018 were evaluated in this retrospective analysis. Exclusion criteria were: bicuspid aortic valve anatomy, transapical TAVI or a valve-in-valve procedure, or the lack of echocardiographic data at baseline and/or follow-up. All patients underwent standard routine transthoracic echocardiography. Demographic and clinical characteristics at the time of TAVI were obtained from the electronic patient records (EPD-vision, Leiden University Medical Center, Leiden, The Netherlands and HiX version 6.1, ChipSoft B.V., Amsterdam, The Netherlands). Clinical characteristics included cardiac risk factors, comorbidities, previous coronary revascularization, and medication use. The estimated glomerular filtration rate (eGFR) was calculated using the Chronic Kidney Disease Epidemiology Collaboration (CKD-EPI) formula, and renal impairment was defined as eGFR < 60 mL/min/1.73 m^2^. This retrospective analysis complies with the Declaration of Helsinki and was approved by the institutional review board which waived the need for written informed consent due to the retrospective study design.

### Transcatheter aortic valve procedure

TAVI eligibility and feasibility as well as decision-making on the access route and valve type were decided by the local heart team. Transcatheter heart valve size was selected based on multidetector-row computed tomography measurements of the aortic annulus, as previously described [20]. The TAVI procedure was performed according to standard practice [21]. Balloon- and self-expandable valves that were used include Edwards SAPIEN, SAPIEN XT, SAPIEN 3 (Edwards Lifesciences, Irvine, CA, USA) and Medtronic CoreValve Evolut R and Evolut Pro (Medtronic, MN, Minnesota, USA).

### Echocardiography

Transthoracic 2-dimensional echocardiographic examinations were performed with patients at rest and the data were acquired from the parasternal, apical, and subcostal views. All echocardiographic examinations were acquired by experienced echocardiographers using commercially available ultrasound systems (Vivid-7, E9 or E95, General Electric Vingmed, Horten, Norway) equipped with 3.5 MHz or M5S transducers. All images including two-dimensional, M-mode, and Doppler data were digitally stored for offline analysis using commercially available software (EchoPac version 113.0.3 and 203; GE Medical Systems, Horten, Norway). The echocardiographic examinations were performed before TAVI (aortic valve hemodynamics, LV function, LV dimensions) and during routine clinical follow-up: immediately after TAVI (prosthetic valve gradients) and at 6 and 12 months after TAVI (prosthetic valve gradients, LV function, LV dimensions), and were reported according to current recommendations [22, 23]. LV dimensions (end-diastolic diameter, intraventricular septum thickness, posterior wall thickness) were obtained in parasternal long-axis views at end-diastole. LV mass was calculated using the Devereux formula and was indexed to body surface area [23]. In addition, the LV remodeling patterns were determined as defined by the relative wall thickness and the presence of LV hypertrophy (defined by a LV mass index > 95 g/m^2^ in women and > 115 g/m^2^ in men): normal geometry (absence of LV hypertrophy and relative wall thickness ≤ 0.42), concentric remodeling (absence of LV hypertrophy and relative wall thickness > 0.42), concentric hypertrophy (LV hypertrophy and relative wall thickness > 0.42), and eccentric hypertrophy (LV hypertrophy and relative wall thickness ≤ 0.42) [23, 24]. AS severity was determined before TAVI. Aortic valve area was calculated using the continuity equation and indexed to body surface area (AVAi). Severe aortic valve stenosis was defined as an aortic valve area < 1.0 cm^2^ or AVAi < 0.6 cm^2^/m^2^, mean transvalvular pressure gradient ≥ 40 mmHg, and a peak aortic jet velocity ≥ 4 m/s [25]. Peak and mean transvalvular gradients were calculated from continuous wave Doppler recordings of the apical 3- or 5-chamber views according to Bernoulli equation [22]. LV volumes (end-diastolic and end-systolic) were measured in the apical 2- and 4-chamber views and indexed to body surface area [23]. Body surface area was considered consistent between each follow-up visit. LVEF was estimated using Simpsons’ biplane method [23]. The presence of post-procedural aortic regurgitation and paravalvular leakage were detected and severity was graded according to current recommendations: mild (grade 1), moderate (grade 2), moderate to severe (grade 3), and severe (grade 4) [26]. Significant paravalvular leakage was defined by a grade ≥ 2.

### Clinical endpoints and follow-up

Changes in prosthetic valve gradients, LV function, and LV mass index over time are presented in absolute numbers and expressed as percentual reduction compared to baseline (pre-TAVI). Additional echocardiographic endpoints to identify sex-related differences in the magnitude of LV reverse remodeling and abnormal prosthetic valve gradients during 1-year echocardiographic follow-up included: (a) ≥ 10% reduction in LV end-diastolic volume index (LVEDVi), (b) ≥ 10% reduction in LV end-systolic volume index (LVESVi), (c) ≥ 10% LV mass index reduction, (d) ≥ 20% LV mass index reduction, (e) mean gradient ≥ 20 mmHg, and (f) reduction in LVEF ≥ 5%.

Patients were followed up for the occurrence of the primary endpoint of all-cause mortality. Survival time was recorded from the final follow-up echocardiographic examination at 6 or 12 months after TAVI and was restricted to 6 years. Data on mortality were collected from the departmental electronic patient files which were linked with the Social Security Death Index. Follow-up data were acquired for all patients.

### Statistical analysis

Continuous variables following a normal distribution are presented as mean ± standard deviation and were compared using the independent Student t-test. Non-normally distributed continuous variables are presented as median with 25-75% interquartile range and were compared using the Mann-Whitney U test. Distribution of continuous variables was evaluated using histograms and Q–Q plots. Categorical variables are presented as absolute numbers and percentages and were compared using the χ2 test or Fisher’s exact test. General linear models with repeated measures analysis were used to evaluate changes in echocardiographic variables over time and to test differences between men and women over time. The Greenhouse-Geisser correction was used if the sphericity assumption was violated. Additional analyses were performed to correct for the potential confounding effect of age, body mass index, hypertension, diabetes mellitus, coronary artery disease, previous myocardial infarction, concomitant moderate or severe mitral regurgitation or aortic regurgitation, moderate or severe paravalvular leakage, pre-TAVI LVEF, and AVAi on the change of the LV parameters over time, and were included as covariates in the general linear models [27]. Kaplan-Meier curves were generated to estimate the cumulative survival rates of all-cause mortality and the log-rank test was used to compare the survival between men and women. Multivariable Cox proportional hazards regression analysis was used to evaluate the association of sex and percentual LV mass index reduction with all-cause mortality. Potential confounders including age, cardiac risk factors, coronary artery disease, previous myocardial infarction, previous stroke/transient ischemic attack, peripheral vascular disease, chronic obstructive pulmonary disease, chronic kidney disease, significant paravalvular leakage, pre-TAVI LVEF, and pre-TAVI LV mass index were incorporated in the multivariable Cox proportional hazard model. Additional multivariable Cox proportional hazard regression models were used to test the association between percentual LV mass index reduction and sex (as interaction term) on outcomes. Hazard ratios (HR) and 95% confidence intervals (CI) were calculated and reported. A two-sided p-value < 0.05 was considered significant. Data analysis was performed with SPSS version 25.0 (IBM SPSS Statistics, IBM Corporation, Armonk, New York, USA).

## Results

### Patient and procedural characteristics

A total of 459 patients (age 80 ± 7 years, 52% males) with severe AS who underwent transfemoral TAVI and had echocardiographic follow-up were included in the analysis. Baseline (pre-TAVI) demographic and clinical characteristics of the overall population and according to sex are presented in Table 1. Women were slightly but significantly older compared to men at the time of TAVI. Men had more frequent history of hypercholesterolemia and concomitant coronary artery disease as well as previous myocardial infarction and coronary artery bypass grafting. Accordingly, men were more frequently using statins and antiplatelet therapy.

**Table 1:** Baseline demographical and clinical characteristics of the overall population and according to sex

Variable	Overall population n = 459	Men n = 240	Women n = 219	p-value
Age, years	80 ± 7	80 ± 7	81 ± 7	0.006
Body surface area, m^2^	1.87 ± 0.21	1.98 ± 0.17	1.74 ± 0.16	< 0.001
Body mass index, kg/m^2^	26.7 ± 4.6	26.8 ± 3.9	26.6 ± 5.3	0.66
Logistic EuroSCORE	12.6 [8.6–20.2]	15.5 [9.0–24.8]	14.5 [10.1–22.3]	0.85
eGFR (CKD-EPI), ml/min/1.73 m^2^	60 ± 20	60 ± 21	61 ± 19	0.66
Hypertension, n (%)	322 (73)	175 (73)	157 (72)	0.83
Hypercholesterolemia, n (%)	278 (61)	156 (65)	122 (56)	0.048
Diabetes mellitus, n (%)	138 (30)	80 (33)	58 (27)	0.11
History of smoke habit, n (%)	67 (15)	41 (17)	26 (12)	0.12
CAD, n (%)	296 (65)	178 (74)	118 (54)	< 0.001
Previous myocardial infarction, n (%)	87 (19)	58 (24)	29 (13)	0.003
Previous revascularization, n (%)				
PCI	143 (31)	80 (33)	63 (29)	0.29
CABG	100 (22)	76 (32)	24 (11)	< 0.001
NYHA classification, n (%)				
I–II	168 (37)	94 (39)	74 (34)	0.23
III–IV	291 (63)	146 (61)	145 (66)	
Previous stroke/TIA, n (%)	87 (19)	44 (19)	43 (20)	0.74
Peripheral vascular disease, n (%)	92 (20)	54 (23)	38 (17)	0.17
Atrial fibrillation, n (%)	114 (25)	68 (28)	46 (21)	0.070
Chronic obstructive pulmonary disease, n (%)	88 (19)	50 (21)	38 (17)	0.35
Medication, n (%)				
Beta-blocker	272 (60)	142 (59)	130 (60)	0.87
ACE-I/ARB II	239 (52)	120 (50)	119 (55)	0.30
Calcium antagonist	107 (23)	60 (25)	47 (22)	0.40
Diuretics	255 (56)	129 (54)	126 (58)	0.35
MR antagonist	50 (11)	35 (15)	15 (7)	0.009
Statins	285 (62)	167 (70)	118 (54)	0.001
Antiplatelet	275 (60)	159 (66)	116 (53)	0.004
Anticoagulation	169 (38)	97 (41)	72 (34)	0.13

Data are presented as mean ± SD, median [25–75% interquartile range] and n (%)

*ACE-I*  angiotensin-converting enzyme, *ARB II* angiotensin-II receptor blocker, *CABG* coronary artery bypass grafting, *CAD* coronary artery disease, *CKD-EPI* chronic kidney disease epidemiology collaboration, *eGFR* estimated glomerular filtration rate, *MR* mineralocorticoid receptor, *NYHA* New York Heart Association, *PCI* percutaneous coronary intervention, *TIA* transient ischemic attack

Pre-TAVI echocardiographic data are presented in Table 2. There were no significant differences between men and women regarding the echocardiographic parameters that define severe AS. LV volumes were significantly larger in men versus women. In addition, LV mass index was significantly larger in men versus women before TAVI (125 ± 32 g/m^2^ vs. 112 ± 33 g/m^2^, respectively, p < 0.001). Concentric hypertrophy was the most frequently observed LV remodeling pattern before TAVI (47%), followed by concentric remodeling (29%), eccentric hypertrophy (16%), and normal geometry (8%), and the distribution of the LV remodeling patterns was comparable between the sexes.

**Table 2:** Echocardiographic data before transcatheter aortic valve implantation

Variable	Overall population n = 459	Men n = 240	Women n = 219	p-value
AV peak gradient, mmHg	65 ± 24	63 ± 22	68 ± 26	0.047
AV mean gradient, mmHg	41 ± 16	41 ± 15	42 ± 17	0.36
AVAi, cm^2^/m^2^	0.41 ± 0.12	0.41 ± 0.14	0.41 ± 0.13	0.72
LVEDVi, ml/m^2^	43 [36–55]	48 [39–59]	39 [33–50]	< 0.001
LVESVi, ml/m^2^	20 [16–29]	22 [17–31]	17 [14–25]	< 0.001
SVi, ml/m^2^	39 ± 13	39 ± 13	38 ± 12	0.40
LVEF, %	51 ± 10	50 ± 10	52 ± 11	0.065
LV mass index, g/m^2^	119 ± 33	125 ± 32	112 ± 33	< 0.001
LVEDD, mm	47.6 ± 7.3	49.9 ± 7.0	45.2 ± 7.0	< 0.001
IVST, mm	13.2 ± 1.8	13.5 ± 1.8	13.0 ± 1.8	0.001
PWT, mm	12.1 ± 2.2	12.5 ± 2.1	11.7 ± 2.1	< 0.001
RWT	0.51 ± 0.13	0.50 ± 0.12	0.52 ± 0.14	0.13
LV remodeling pattern				
Normal geometry	37 (8)	24 (10)	13 (6)	0.19
Concentric remodeling	133 (29)	70 (29)	63 (29)	
Concentric hypertrophy	215 (47)	113 (48)	102 (46)	
Eccentric hypertrophy	72 (16)	31 (13)	41 (19)	

Data are presented as mean ± SD, median [25–75% interquartile range] and n (%)

*AV* aortic valve, AVAi = indexed aortic valve area, *IVST* intraventricular septum thickness, *LV* left ventricular, *LVEDVi* left ventricular end-diastolic volume index, *LVEDD* left ventricular end-diastolic diameter, *LVEF* left ventricular ejection fraction, *LVESVi* left ventricular end-systolic volume index, *PWT* posterior wall thickness, *RWT* relative wall thickness, *SVi* stroke volume index

### Left ventricular and aortic valve changes after TAVI

All patients underwent TAVI via the transfemoral approach. The majority of patients received balloon-expandable valves: Edwards SAPIEN 3 (57%), SAPIEN XT (10%), and SAPIEN (8%). Self-expandable valves were used in 19% of patients. Prosthesis size ranged from 20 to 31 mm, with 26 mm being most frequently used in 183 patients (40%).

**Fig. 1:**
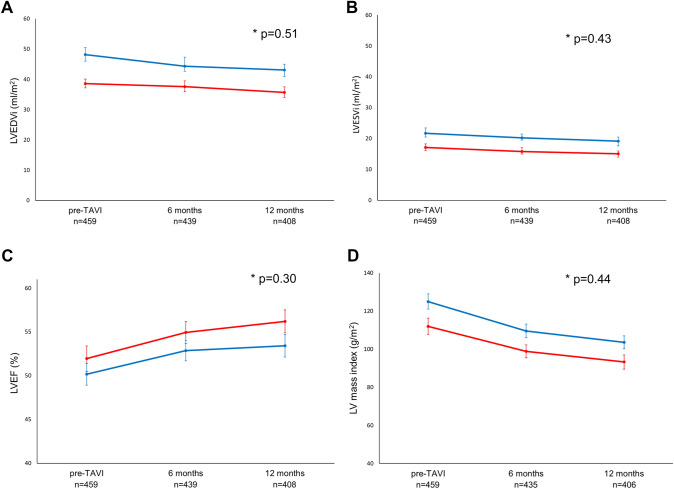
Left ventricular reverse remodeling in men (blue) and women (red) after transcatheter aortic valve implantation. Changes in left ventricular end-diastolic volume index (LVEDVi, panel **A**), left ventricular end-systolic volume index (LVESVi, panel **B**), left ventricular ejection fraction (LVEF, panel **C**), and left ventricular mass index (panel **D**) from baseline to 6 months and 12 months follow-up after transcatheter aortic valve implantation. * shows p-value for interaction between men and women over time. Error bars indicate 95% confidence intervals

**Fig. 2:**
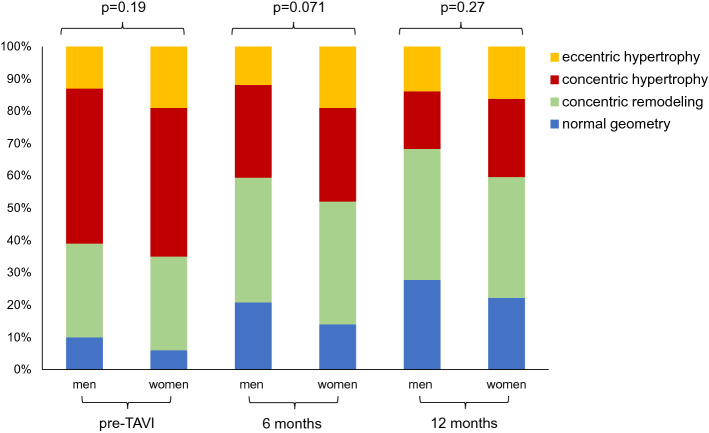
Distribution of the left ventricular remodeling patterns among men and women before transcatheter aortic valve implantation and at 6 and 12 months follow-up

Changes in LV volumes and LV mass index as well as LVEF over time, are displayed in Fig. 1. At 6 and 12 months after TAVI, both men and women showed a significant reduction in LVEDVi (from 48 [39–59] ml/m^2^ to 44 [37–55] ml/m^2^ at 6 months and to 43 [34–53] ml/m^2^ at 12 months after TAVI; and from 39 [33–50] ml/m^2^ to 38 [31–46] ml/m^2^ at 6 months and to 36 [28–43] ml/m^2^ at 12 months, respectively, both p < 0.001) and in LVESVi (men: from 22 [17–31] ml/m^2^ to 20 [16–27] ml/m^2^ at 6 months and to 19 [15–26] ml/m^2^ at 12 months after TAVI; women: from 17 [14–25] ml/m^2^ to 16 [13–21] ml/m^2^ at 6 months and to 15 [15–20] ml/m^2^ at 12 months, respectively, both p < 0.001), without significant differences between the sexes over time (p for interaction = 0.51 and 0.43, respectively). Moreover, LV mass index regressed significantly in both groups during follow-up (men: from 125 ± 32 g/m^2^ to 110 ± 27 g/m^2^ at 6 months and to 104 ± 25 g/m^2^ at 12 months after TAVI, p < 0.001; women: from 112 ± 33 g/m^2^ to 99 ± 24 g/m^2^ at 6 months and to 93 ± 26 g/m^2^ at 12 months, p < 0.001; p for interaction = 0.44). Additionally, LVEF improved significantly in both groups during follow-up, without significant differences between men and women over time (from 50 ± 10% to 53 ± 9% at 6 months and to 53 ± 10% at 12 months after TAVI in men, p < 0.001 and from 52 ± 11% to 55 ± 9% at 6 months and to 56 ± 9% at 12 months in women, p < 0.001; p for interaction = 0.30). Similar results were observed when adjusting for potential confounders of LV reverse remodeling: no significant differences between men and women over time were observed in terms of improvement in LVEF and reduction in indexed LV volumes and LV mass. The distribution of the LV remodeling patterns for men and women before TAVI and at 6 and 12 months follow-up are displayed in Fig. 2.

**Fig. 3:**
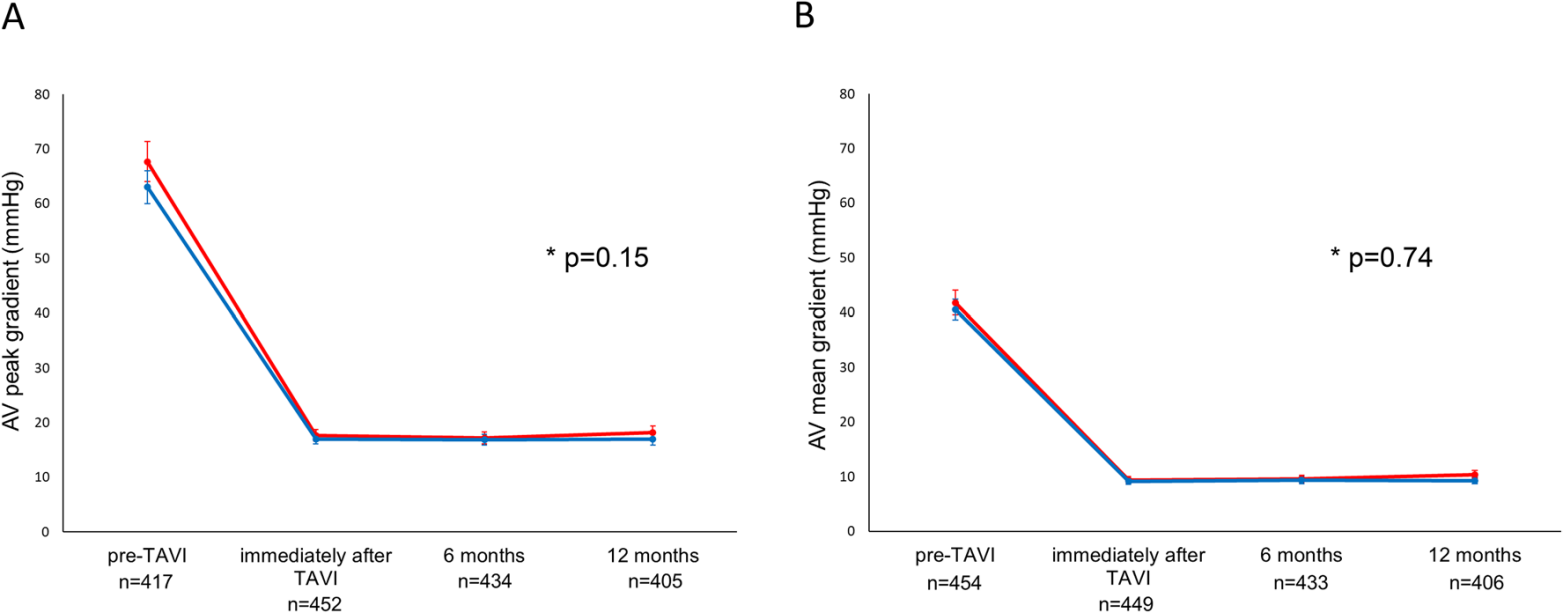
Aortic valve gradients of men (blue) and women (red) before transcatheter aortic valve implantation and during follow-up. Changes in aortic valve peak gradient (panel A) and aortic valve mean gradient (panel B) from baseline to immediately after transcatheter aortic valve implantation and to 6 and 12 months follow-up. * shows p-value for interaction between men and women over time. Error bars indicate 95% confidence intervals

Changes in aortic valve prosthetic valve gradients over time are displayed in Fig. 3. Aortic valve peak gradient decreased significantly after TAVI, without significant differences between men and women over time (men: from 63 ± 22 mmHg to 17 ± 6 mmHg immediately after TAVI and to 17 ± 7 mmHg at 6 months and to 17 ± 8 mmHg at 12 months after TAVI, p < 0.001; women: from 68 ± 26 mmHg to 18 ± 8 mmHg immediately after TAVI and to 17 ± 8 mmHg at 6 months and to 18 ± 8 mmHg at 12 months, p < 0.001, respectively; p for interaction = 0.15). Similarly, aortic valve mean gradients decreased significantly in both groups (men: from 41 ± 15 mmHg to 9 ± 4 mmHg immediately after TAVI and to 9 ± 5 mmHg at 6 and 12 months after TAVI, p < 0.001; women: from 42 ± 17 mmHg to 9 ± 5 mmHg immediately after TAVI and to 10 ± 5 mmHg at 6 and 12 months, p < 0.001; p for interaction = 0.74). The echocardiographic data of the overall population and according to sex at 6 months and 12 months follow-up are summarized in Supplemental Table 1.

The echocardiographic endpoints for the overall population and according to sex at 6 and 12 months after TAVI are summarized in Table 3. No significant differences were observed between men and women in terms of absolute reduction of indexed LV volumes and LV mass as well as improvement in LV function, nor if expressed as percentual change as compared to baseline. Moreover, a ≥ 10% reduction in indexed LV volumes and LV mass during follow-up was equally observed among men and women. In addition, significant paravalvular leakage was noted in 27 (6%) patients during 12 months follow-up after TAVI and was comparable between men and women (5% vs. 6%, respectively, p = 0.66). The rates of increased aortic valve prosthetic mean gradient (≥ 20 mmHg) were comparable between men and women at 6 months after TAVI, but were more common in women at 12 months follow-up (6% vs. 2%, respectively, p = 0.041).

**Table 3:** Echocardiographic endpoints at 6 months and 12 months after transcatheter aortic valve implantation

Variable		Overall population	Men	Women	p-value
Absolute LVEDVi reduction, ml/m^2^	6 m (n = 439)	1.7 [− 3.8–8.5]	1.6 [− 3.6–10.0]	1.8 [− 3.8–7.1]	0.50
	12 m (n = 408)	4.4 [− 2.9–10.8]	4.4 [− 3.2–10.9]	4.4 [− 2.0–10.5]	0.74
Absolute LVESVi reduction, ml/m^2^	6 m (n = 439)	1.5 [− 1.3–5.7]	1.9 [− 1.3–6.5]	1.2 [− 1.3–4.4]	0.25
	12 m (n = 408)	2.9 [− 0.5–7.5]	2.9 [− 1.1–7.7]	3.0 [− 0.3–7.0]	0.58
Absolute LVEF improvement, %	6 m (n = 439)	2.3 [− 2.8–8.3]	2.2 [− 2.8–7.7]	2.3 [− 2.7–8.7]	0.69
	12 m (n = 408)	3.4 [− 1.7–8.5]	3.0 [− 2.6–7.7]	4.2 [− 1.0–9.1]	0.15
Absolute LV mass index reduction, g/m^2^	6 m (n = 435)	12 [0–25]	12 [1–27]	12 [− 1–23]	0.24
	12 m (n = 406)	17 [6–31]	17 [7–32]	17 [5–31]	0.78
LVEDVi reduction as percentage of baseline, %	6 m (n = 439)	4.0 [− 10.5–18.2]	4.0 [− 8.0–19.3]	4.4 [− 11.5–17.6]	0.67
	12 m (n = 408)	9.3 [− 6.9–24.3]	8.9 [− 7.4–23.1]	9.4 [− 5.6–23.9]	0.44
LVESVi reduction as percentage of baseline, %	6 m (n = 439)	7.7 [− 6.7–24.5]	7.7 [− 5.3–24.5]	7.4 [− 7.2–23.9]	0.63
	12 m (n = 408)	15.8 [− 2.9–33.2]	14.3 [− 3.6–31.4]	16.8 [− 1.9–36.0]	0.21
LV mass index reduction as percentage of baseline, %	6 m (n = 435)	10.7 [0–20.6]	10.5 [0.5–20.4]	10.9 [− 1.6–21.1]	0.71
	12 m (n = 406)	15.5 [5.8–25.4]	15.0 [5.7–23.6]	17.0 [5.7–26.7]	0.42
LVEDVi reduction, n (%)	6 m (n = 439)	261 (60)	137 (59)	124 (60)	0.95
	12 m (n = 408)	269 (66)	138 (64)	131 (68)	0.36
LVESVi reduction, n (%)	6 m (n = 439)	288 (66)	154 (67)	134 (64)	0.62
	12 m (n = 408)	283 (69)	145 (67)	138 (72)	0.30
LVEF improvement, n (%)	6 m (n = 439)	274 (62)	138 (60)	136 (65)	0.22
	12 m (n = 408)	280 (69)	143 (66)	137 (71)	0.26
LV mass index reduction, n (%)	6 m (n = 435)	324 (75)	175 (77)	149 (72)	0.25
	12 m (n = 406)	337 (83)	179 (84)	158 (82)	0.56
≥ 10% LVEDVi reduction, n (%)	6 m (n = 439)	178 (41)	94 (41)	84 (40)	0.95
	12 m (n = 408)	200 (49)	105 (49)	95 (50)	0.86
≥ 10% LVESVi reduction, n (%)	6 m (n = 439)	201 (46)	109 (47)	92 (44)	0.54
	12 m (n = 408)	234 (57)	119 (55)	115 (60)	0.33
≥ 10% LV mass index reduction, n (%)	6 m (n = 435)	231 (53)	122 (54)	109 (53)	0.86
	12 m (n = 406)	271 (67)	144 (68)	127 (66)	0.70
≥ 20% LV mass index regression, n (%)	6 m (n = 435)	117 (27)	61 (27)	56 (27)	0.94
	12 m (n = 406)	150 (37)	71 (33)	79 (41)	0.11
AV mean gradient ≥ 20 mmHg, n (%)	6 m (n = 434)	15 (4)	6 (3)	9 (4)	0.33
	12 m (n = 406)	15 (4)	4 (2)	11 (6)	0.041
≥ 5% LVEF reduction, n (%)	6 m (n = 439)	70 (16)	35 (15)	35 (17)	0.63
	12 m (n = 408)	62 (15)	34 (16)	28 (15)	0.75

Data are presented as mean ± SD and n (%)

*AV* aortic valve, *LV* left ventricular, *LVEDVi* left ventricular end-diastolic volume index, *LVEF* left ventricular ejection fraction, *LVESVi* left ventricular end-systolic volume index

### Clinical outcomes and impact of sex on LV reverse remodeling

**Fig. 4:**
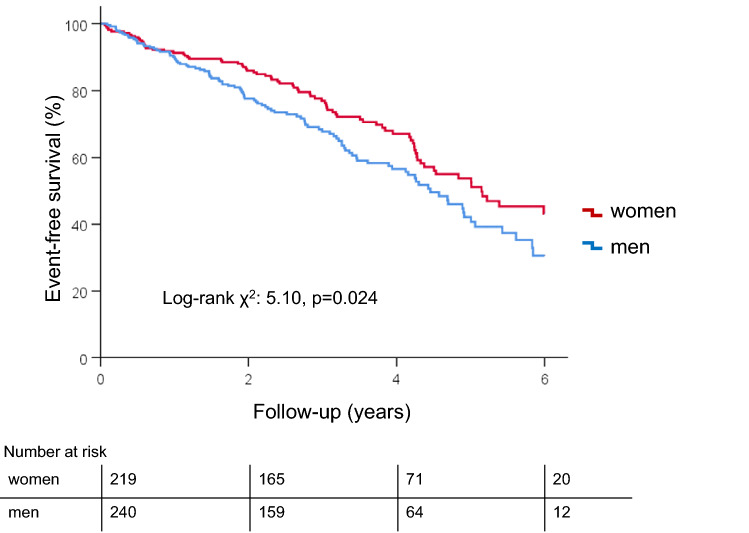
Kaplan-Meier curves demonstrating the event-free survival for all-cause mortality according to sex

During a median follow-up of 2.8 [IQR 1.9–4.3] years after the 6 or 12 months echocardiographic examination following TAVI, 181 (39%) patients died of which 103 were men and 78 women. Overall, the cumulative survival rates were 90% at 1 year and 46% at 5 years. Kaplan-Meier analysis demonstrated superior long-term survival in women versus men (log-rank χ^2^: 5.10, p = 0.024, Fig. 4). Moreover, multivariable Cox proportional hazard regression analysis demonstrated that male sex was independently associated with an increased risk of all-cause mortality after adjusting for age, cardiac risk factors, comorbidities, significant paravalvular leakage, pre-TAVI LVEF, and pre-TAVI LV mass index (HR 1.408, 95% CI 1.030–1.923, p = 0.032, Table 4). In addition, there was no significant association between the percentual LV mass index reduction and sex (as an interaction term) versus outcomes (p = 0.64).

**Table 4:** Uni- and multivariable Cox regression analysis for all-cause mortality

Parameter	Univariable			Multivariable		
	HR	95% CI	p-value	HR	95% CI	p-value
Male sex	1.402	1.044–1.883	0.025	1.408	1.030–1.923	0.032
1% LV mass index reduction	0.992	0.984–1.000	0.065	0.992	0.982–1.002	0.097

Adjusted for the following: age, hypertension, hypercholesterolemia, diabetes mellitus, smoking, coronary artery disease, previous myocardial infarction, previous stroke/transient ischemic attack, peripheral vascular disease, chronic obstructive pulmonary disease, chronic kidney disease, significant paravalvular leakage, pre-TAVI left ventricular ejection fraction, and pre-TAVI left ventricular mass index

## Discussion

In patients with severe AS, men and women showed a similar reduction in LV volumes and LV mass as well as improvement in LVEF after TAVI, despite smaller LV volumes and mass in women before TAVI. In addition, women showed better survival after TAVI as compared to men. However, the survival benefit of women is not explained by sex differences in LV reverse remodeling after TAVI.

### Sex differences in LV reverse remodeling after TAVI

AS causes an increase in LV afterload to which the myocardium responds with LV hypertrophy in order to normalize wall stress and maintain cardiac output [15, 28]. Aortic valve replacement directly relieves the increased LV afterload, leading to lower aortic valve gradients and LV reverse remodeling with LV mass regression, reduction in LV volumes, and improvement in LV systolic function [29, 30]. Similarly, the current study showed that patients with severe AS had a significant reduction in LV volumes and LV mass with an improvement of LVEF during the first year after TAVI.

Sex-related differences in LV remodeling in response to the increased pressure overload caused by AS have been noted. Before aortic valve replacement, women show smaller LV volumes and LV mass as compared to men for a similar degree of AS severity [28, 31]. However, at follow-up after TAVI there is a similar magnitude of reduction in LV mass index and volumes and improvement in LVEF in men and women, as shown in the present study. Similarly, a study including 100 patients with severe AS treated with surgical aortic valve replacement or TAVI did not show sex differences in LV reverse remodeling at 6 months using cardiac magnetic resonance imaging [16]. In contrast, a study evaluating 100 patients with severe AS treated with TAVI reported a significant improvement in LVEF among women at 3 months after TAVI, but not in men [17]. In addition, Ninomiya et al. [32] showed in a study of 208 patients with severe AS treated with TAVI, that the incidence of LV reverse remodeling (defined as a reduction of LV end-systolic volume > 15% evaluated by echocardiography at 3 months after TAVI) was significantly higher in men versus women. These results show the heterogeneity in defining LV reverse remodeling after TAVI which challenges the comparison across studies.

### Sex differences in outcomes after TAVI and association with LV reverse remodeling

This study demonstrated superior survival in women after TAVI in line with previous reports [10, 11]. Several factors have been suggested for the sex-related differences in outcomes after TAVI, including sex differences in (paravalvular) aortic regurgitation of the transcatheter valve due to patient-prothesis mismatch, more co-existent comorbidities in men, the timing of referral/treatment of patients with severe AS (women earlier referral than men), and the longer life expectancy in women versus men [12].

Another explanation may be the sex-specific myocardial remodeling in AS. Previously, our group examined the impact of sex on LV remodeling before TAVI and reported that the outcome benefits of women after TAVI were not associated with sex differences in LV remodeling [33]. However, the impact of the potential sex-related differences in LV reverse remodeling after TAVI on outcomes remained unexplored. In the current study, no significant interaction was observed between sex and LV reverse remodeling and its association with outcomes. It is important to note that men remained with larger LV volumes and LV mass index as compared to women at follow-up after TAVI, which could indicate that men have a more pronounced LV hypertrophic response to severe AS that is not fully recovered when the pressure overload is relieved. This might be related to the observed increased risk for all-cause mortality [34]. However, the interaction term of sex and the magnitude in reduction of LV mass index and LV volumes was not independently associated with all-cause mortality.

### Future studies

Further research is necessary to confirm our observations and to elucidate the potential causes of the sex differences in outcomes observed after aortic valve replacement. Currently, the prospective, multicenter, Randomized researcH in womEn all comers wIth Aortic stenosis (RHEIA) trial is being performed to evaluate the safety and efficacy of surgical aortic valve replacement versus TAVI specifically in women with severe AS [35]. The results of the RHEIA trial may provide important data for the optimal therapeutic management of severe AS in women.

### Limitations

Several limitations should be acknowledged. This is a single-center retrospective analysis with inherent limitations related to the study design. Second, only patients with echocardiographic follow-up after TAVI were included in the current analysis which may have introduced a selection bias. In addition, the echocardiographic follow-up was limited to 1 year, whereas LV reverse remodeling is a process that may continue beyond 1 year after aortic valve replacement. Last, arterial afterload (i.e. co-existent arterial hypertension) and myocardial fibrosis may affect LV reverse remodeling after TAVI. However, this study used echocardiography to assess LV reverse remodeling and could detect the presence and extent of myocardial fibrosis.

## Conclusions

Patients with severe AS had a significant improvement in LVEF, reduction in LV volumes and LV mass index at 6 and 12 months after TAVI, without significant differences between men and women over time. Women showed better survival after TAVI as compared to men. However, the interaction between the percentual LV mass index reduction and sex was not associated with survival. These data suggest that the superior outcomes noted in women after TAVI are not associated with sex differences in LV reverse remodeling.

### Supplementary Information

Below is the link to the electronic supplementary material.

Supplementary material 1 (DOCX 17.0 kb)

## Data Availability

The data underlying this article will be shared on reasonable request to the corresponding author.
