# Supplementary material for: Interaction between sex and left ventricular reverse remodeling and its association with outcomes after transcatheter aortic valve implantation
Source: Int J Cardiovasc Imaging. 2022 Apr 21;38(9):1973–85. doi: 10.1007/s10554-022-02596-x (PMC10509071; doi:10.1007/s10554-022-02596-x)
Supplement: Supplementary file 1 — Supplementary material 1 (DOCX 17.0 kb) [file 10554_2022_2596_MOESM1_ESM.docx]

**SUPPLEMENTAL DATA**

**Supplemental Table 1.** Echocardiographic data at 6 months and 12 months after transcatheter aortic valve implantation.

|  | 6 months after TAVI | | | | 12 months after TAVI | | | |
| --- | --- | --- | --- | --- | --- | --- | --- | --- |
| Variable | **Overall population n=439** | **Men n=231** | **Women n=208** | **p-value** | **Overall population n=408** | **Men n=216** | **Women n=192** | **p-value** |
| AV peak gradient, mmHg | 17 ± 8 | 17 ± 7 | 17 ± 8 | 0.66 | 18 ± 8 | 17 ± 8 | 18 ± 8 | 0.13 |
| AV mean gradient, mmHg | 10 ± 5 | 9 ± 5 | 10 ± 5 | 0.68 | 10 ± 5 | 9 ± 5 | 10 ± 5 | 0.028 |
| LVEDVi, ml/m^2^ | 42 [34-51] | 44 [37-55] | 38 [31-46] | <0.001 | 39 [31-49] | 43 [34-53] | 36 [28-43] | <0.001 |
| LVESVi, ml/m^2^ | 18 [14-24] | 20 [16-27] | 16 [13-21] | <0.001 | 17 [13-24] | 19 [15-26] | 15 [11-20] | <0.001 |
| LVEF, % | 54 ± 9 | 53 ± 9 | 55 ± 9 | 0.019 | 55 ± 10 | 53 ± 10 | 56 ± 9 | 0.003 |
| LV mass index, g/m^2^ | 105 ± 26 | 110 ± 27 | 99 ± 24 | <0.001 | 99 ± 26 | 104 ± 25 | 93 ± 26 | <0.001 |
| LVEDD, mm | 46.5 ± 7.2 | 48.7 ± 7.1 | 44.0 ± 6.6 | <0.001 | 46.3 ± 6.9 | 48.8 ± 6.4 | 43.6 ± 6.5 | <0.001 |
| IVST, mm | 12.7 ± 1.7 | 12.9 ± 1.8 | 12.5 ± 1.7 | 0.036 | 12.5 ± 1.7 | 12.7 ± 1.7 | 12.3 ± 1.7 | 0.036 |
| PWT, mm | 11.3 ± 2.0 | 11.6 ± 2.0 | 10.9 ± 2.0 | <0.001 | 10.7 ± 1.9 | 11.0 ± 1.9 | 10.4 ± 1.8 | 0.004 |
| RWT | 0.48 ± 0.12 | 0.47 ± 0.12 | 0.50 ± 0.13 | 0.071 | 0.46 ± 0.12 | 0.44 ± 0.11 | 0.48 ± 0.13 | 0.011 |
| LV remodeling pattern |  |  |  |  |  |  |  |  |
| Normal geometry | 77 (18) | 48 (21) | 29 (14) | 0.071 | 102 (25) | 59 (28) | 43 (22) | 0.27 |
| Concentric remodeling | 166 (38) | 88 (39) | 78 (38) |  | 159 (39) | 87 (41) | 72 (37) |  |
| Concentric hypertrophy | 125 (29) | 65 (29) | 60 (29) |  | 85 (21) | 38 (18) | 47 (24) |  |
| Eccentric hypertrophy | 67 (15) | 27 (12) | 40 (19) |  | 60 (15) | 29 (14) | 31 (16) |  |

Data are presented as mean ± SD, median [25-75% interquartile range] and n (%). AV = aortic valve, AVAi = indexed aortic valve area, IVST = intraventricular septum thickness, LV = left ventricular, LVEDVi = left ventricular end-diastolic volume index, LVEDD = left ventricular end-diastolic diameter, LVEF = left ventricular ejection fraction, LVESVi = left ventricular end-systolic volume index, PWT = posterior wall thickness, RWT = relative wall thickness.
